# Gene Amplification Uncovers Large Previously Unrecognized Cryptic Antibiotic Resistance Potential in E. coli

**DOI:** 10.1128/Spectrum.00289-21

**Published:** 2021-11-10

**Authors:** Stacy A. Suarez, Adam C. Martiny

**Affiliations:** a Department of Ecology and Evolutionary Biology, University of California, Irvine, California, USA; b Department of Earth System Science, University of California, Irvine, California, USA; Hartford Hospital

**Keywords:** adaptive resistance, antibiotic resistance, drug resistance evolution, gene amplification

## Abstract

The activation of unrecognized antibiotic resistance genes in the bacterial cell can give rise to antibiotic resistance without the need for major mutations or horizontal gene transfer. We hypothesize that bacteria harbor an extensive array of diverse cryptic genes that can be activated in response to antibiotics via adaptive resistance. To test this hypothesis, we developed a plasmid assay to randomly manipulate gene copy numbers in Escherichia coli cells and identify genes that conferred resistance when amplified. We then tested for cryptic resistance to 18 antibiotics and identified genes conferring resistance. E. coli could become resistant to 50% of the antibiotics tested, including chloramphenicol, d-cycloserine, polymyxin B, and 6 beta-lactam antibiotics, following this manipulation. Known antibiotic resistance genes comprised 13% of the total identified genes, where 87% were unclassified (cryptic) antibiotic resistance genes. These unclassified genes encoded cell membrane proteins, stress response/DNA repair proteins, transporters, and miscellaneous or hypothetical proteins. Stress response/DNA repair genes have a broad antibiotic resistance potential, as this gene class, in aggregate, conferred cryptic resistance to nearly all resistance-positive antibiotics. We found that antibiotics that are hydrophilic, those that are amphipathic, and those that inhibit the cytoplasmic membrane or cell wall biosynthesis were more likely to induce cryptic resistance in E. coli. This study reveals a diversity of cryptic genes that confer an antibiotic resistance phenotype when present in high copy number. Thus, our assay can identify potential novel resistance genes while also describing which antibiotics are prone to induce cryptic antibiotic resistance in E. coli.

**IMPORTANCE** Predicting where new antibiotic resistance genes will rise is a challenge and is especially important when new antibiotics are developed. Adaptive resistance allows sensitive bacterial cells to become transiently resistant to antibiotics. This provides an opportune time for cells to develop more efficient resistance mechanisms, such as tolerance and permanent resistance to higher antibiotic concentrations. The biochemical diversity harbored within bacterial genomes may lead to the presence of genes that could confer resistance when timely activated. Therefore, it is crucial to understand adaptive resistance to identify potential resistance genes and prolong antibiotics. Here, we investigate cryptic resistance, an adaptive resistance mechanism, and identify unknown (cryptic) antibiotic resistance genes that confer resistance when amplified in a laboratory strain of E. coli. We also pinpoint antibiotic characteristics that are likely to induce cryptic resistance. This study may help detect novel antibiotic resistance genes and provide the foundation to help develop more effective antibiotics.

## INTRODUCTION

The rapid spread and emergence of antibiotic resistance make it one of the major threats to global public health (1, 2). Antibiotic-resistant bacteria annually infect nearly 3 million people in the United States (1) and are projected to cause 10 million human deaths worldwide per year by 2050, more than the current rate for cancer (3). Therefore, it is critical to understand the evolution of antibiotic resistance to effectively tackle this worldwide crisis.

The emergence of antibiotic resistance is generally due to acquired, intrinsic, or adaptive resistance (4). Acquired resistance is the traditional pathway, which includes mutations in chromosomal genes and horizontal gene transfer. Intrinsic resistance refers to the inherent properties (such as efflux pumps) of the bacterial cell that can influence resistance. Intrinsic antibiotic resistance (AR) genes contribute to resistance at the wild-type expression level (5). Adaptive resistance, which includes cryptic resistance, does not have a universally accepted definition (6), but it has been defined as “a temporary increase in the ability of a bacterium to survive antibiotic insult due to alterations in gene and/or protein expression as a result of exposure to an environmental trigger” (4). Contrary to acquired and intrinsic resistance, adaptive resistance is dependent on the antibiotic resulting in an unstable phenotype.

Latent resistance is a form of adaptive resistance, and latent AR genes have the potential to contribute to resistance if their expression is changed from that of the wild type (5). Latent antibiotic resistance may occur by the activation of unclassified (cryptic) AR genes in the bacterial cell (5, 7–10). Cryptic genes can be any gene not commonly known to confer antibiotic resistance. Only recently have studies emerged that thoroughly investigate the link between antibiotic resistance and the amplification of unrecognized AR genes (5, 7–10).

There may be a large potential for an unrecognized and diverse reservoir of latent AR genes in pathogens, as cryptic resistance can occur without major mutation and horizontal transmission. Additionally, the vast biochemical diversity harbored within bacterial genomes furthers the potential for the presence of cryptic genes that could confer resistance when necessary. For example, the method scalar analysis of library enrichments was used to identify genomic regions that, when upregulated, led to cryptic aminoglycoside resistance in Pseudomonas aeruginosa (7). Genes that increased aminoglycoside resistance encoded products related to DNA repair, O-antigen synthesis, and transcriptional and translational processes. Gene expression variability was measured in Escherichia coli adapted to ampicillin, tetracycline, or n-butanol, showing that the top three categories for overexpressed genes were metabolic and biosynthetic processes, membrane components, and response to stimuli (9). A transposon tool, GeneHunter, has also been used to identify cryptic/latent AR genes in Salmonella enterica (10). Recently, intrinsic and latent resistance genes were identified in E. coli via a disk diffusion assay (5). Understanding cryptic resistance is crucial to ultimately reduce the evolution to current and new antibiotics. The molecular mechanisms and types of antibiotics that lead to cryptic resistance are still unclear, but delineating these will further elucidate the emergence of antibiotic resistance.

Adaptive (latent) resistance may provide a link to mutational resistance, which endures in the absence of the antibiotic (6). E. coli adapted to amoxicillin, tetracycline, and enrofloxacin exposure showed that an initial differential gene expression response led to mutations conferring higher antibiotic resistance (11). Adaptive resistance, which is not classified as tolerance or resistance but rather a connection between the two, leads to transient resistance to low antibiotic concentrations for long periods of time. Tolerance, which has shown to facilitate the development of mutational resistance to antibiotics in E. coli (12), allows cells to resist high antibiotic concentrations for short periods of time (6). Adaptive resistance could be an opportune time for bacterial cells to develop more efficient resistance mechanisms, such as tolerance and permanent resistance to higher antibiotic concentrations. Additionally, overexpression of unrecognized AR genes imparts a minor to zero effect on fitness in the absence of the antibiotic (5, 8). In contrast, antibiotic resistance mutations can be costly; for example, fluoroquinolone resistance in pseudomonads can hinder motility (13). Our overarching hypothesis is that bacteria harbor an extensive array of diverse cryptic latent AR genes that will confer resistance when amplified. We predict that these genes will be associated with the antibiotic mechanism of action and that cryptic resistance will be less common in the presence of newer antibiotics due to their stronger activity.

Here, we developed a plasmid assay adapted from functional metagenomics, which incorporates a high-throughput method to determine if a large increase in gene copy number can cause an AR phenotype in E. coli in the absence of chromosomal mutations. We specifically asked the following: (i) what are the genes that confer an AR phenotype when amplified and (ii) which types of antibiotics will induce resistance in this manner? If we find cryptic genes conferring an AR phenotype when amplified, then this may demonstrate a prevalent resistance mechanism, allowing us to identify genes not known to be considered AR genes.

## RESULTS AND DISCUSSION

**FIG 1:**
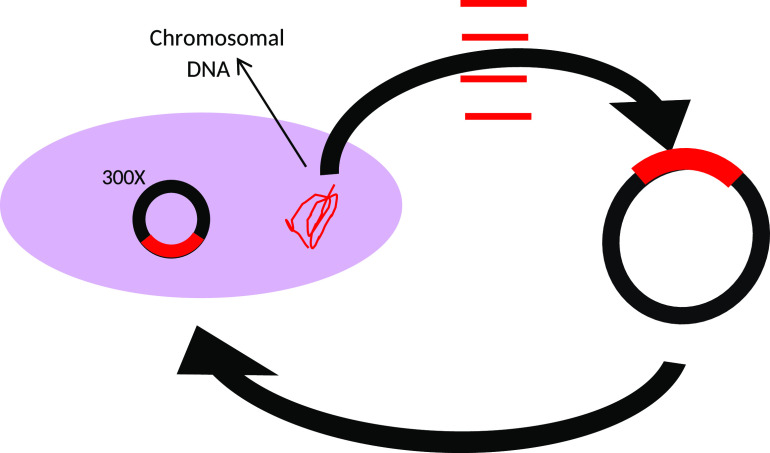
Gene amplification assay we developed to test for latent and cryptic antibiotic resistance.

**FIG 2:**
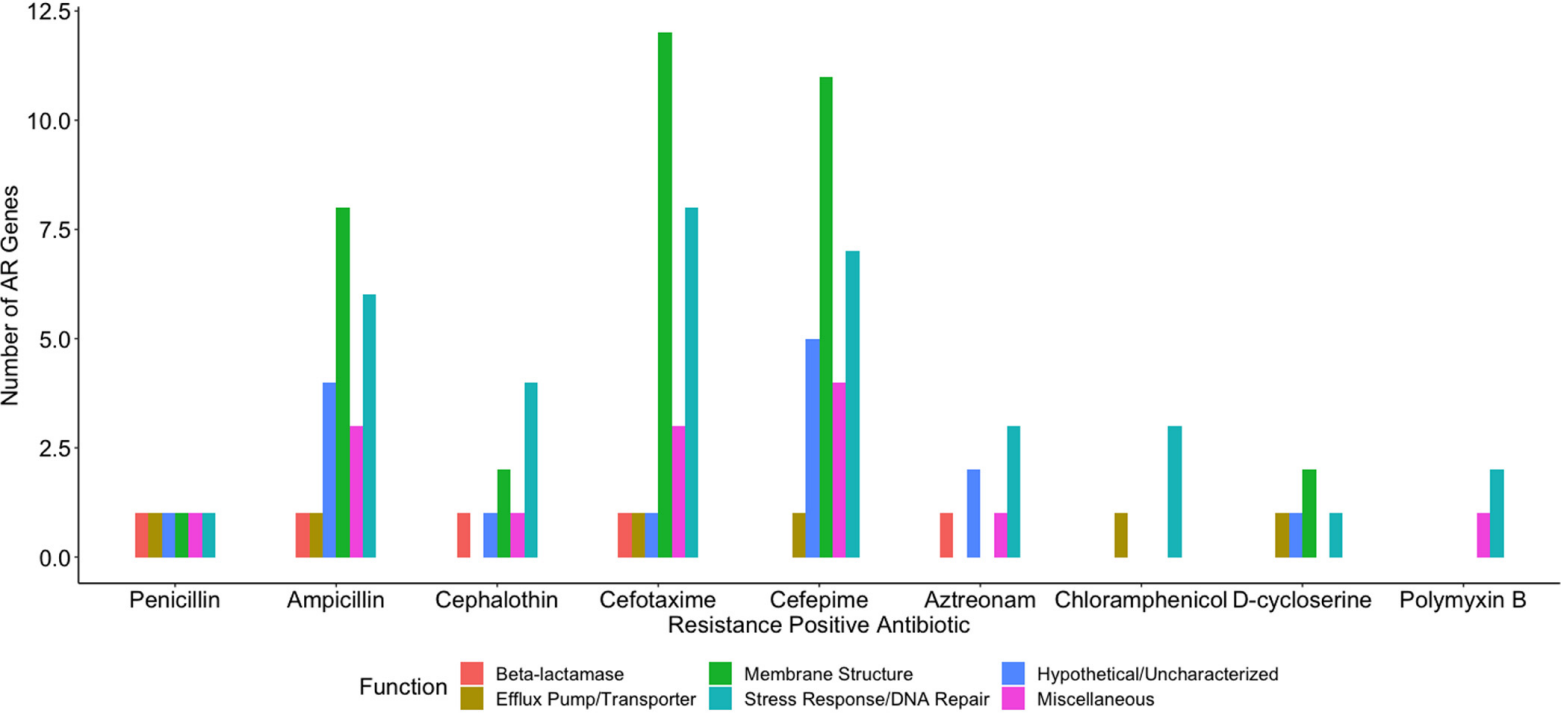
Number of antibiotic resistance genes conferring latent resistance to antibiotics at the MICs. Penicillin-aztreonam are beta-lactams.

**FIG 3:**
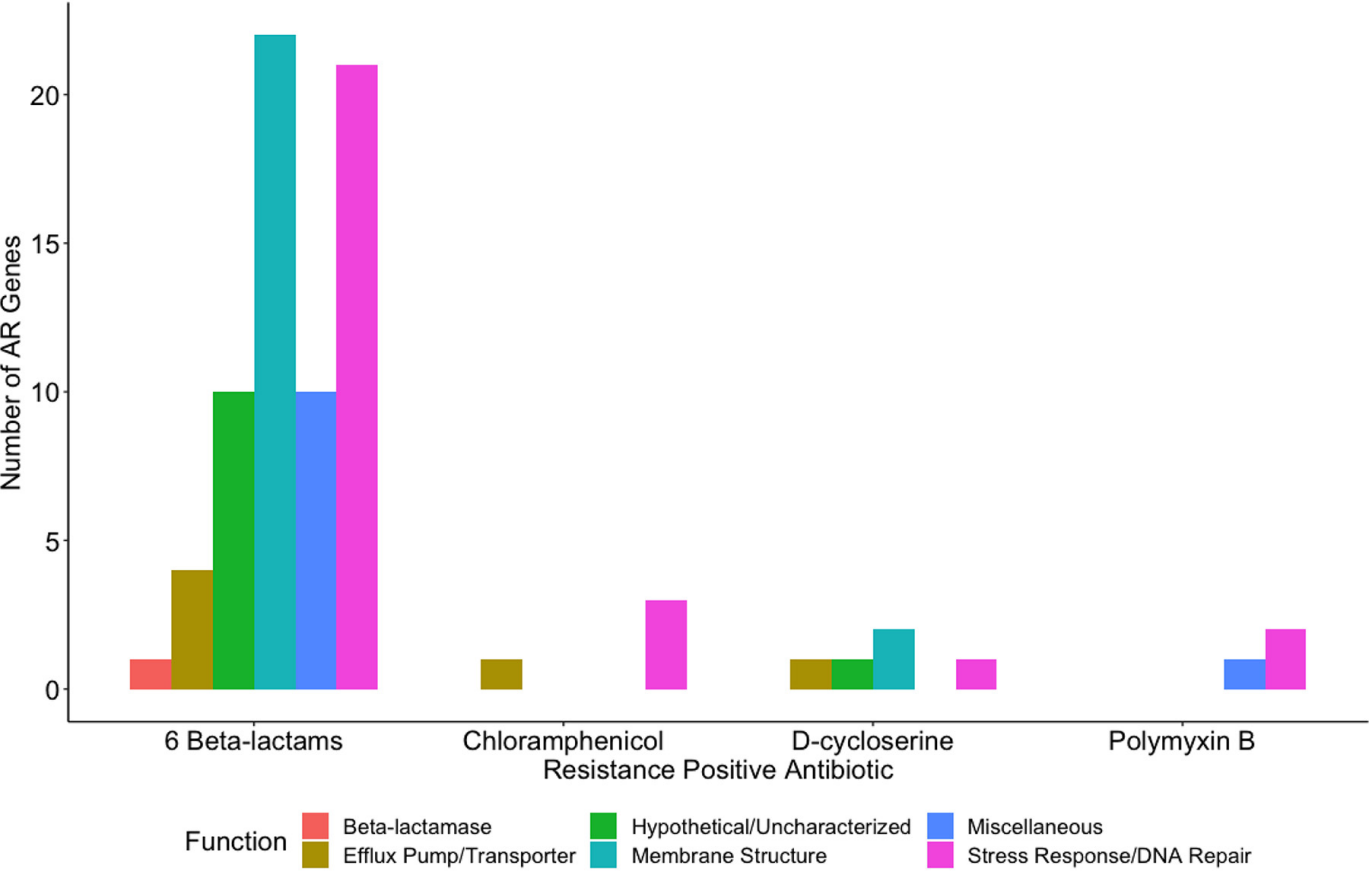
Number of antibiotic resistance genes conferring latent resistance to antibiotics at the MICs, separated by class.

**FIG 4:**
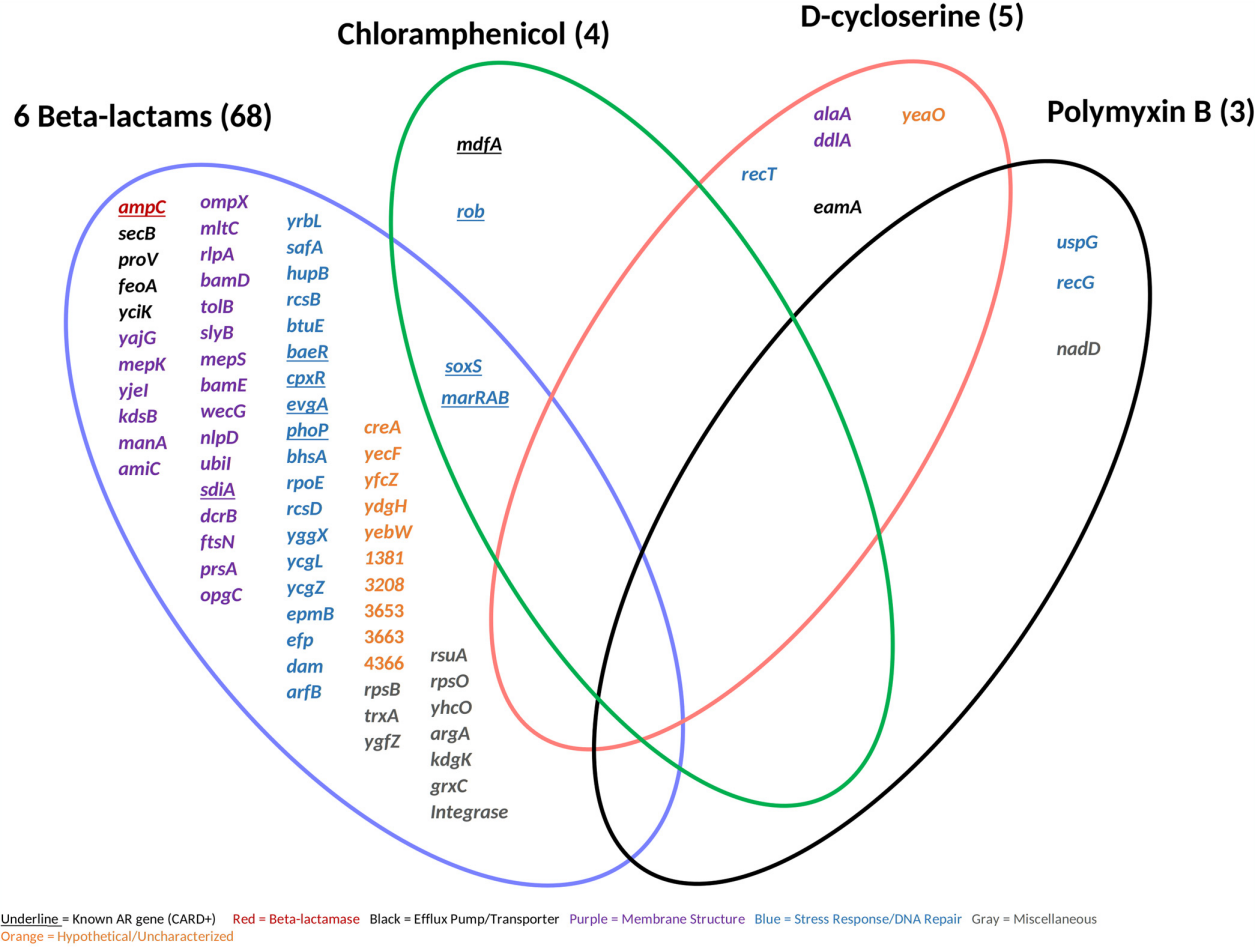
Antibiotic resistance genes shared between all resistance-positive antibiotics (9) separated by class. We identified 78 antibiotic resistance genes (shown) causing resistance at the MICs. Known antibiotic resistance genes were classified using the Comprehensive Antibiotic Resistance Database by gene name.

**FIG 5:**
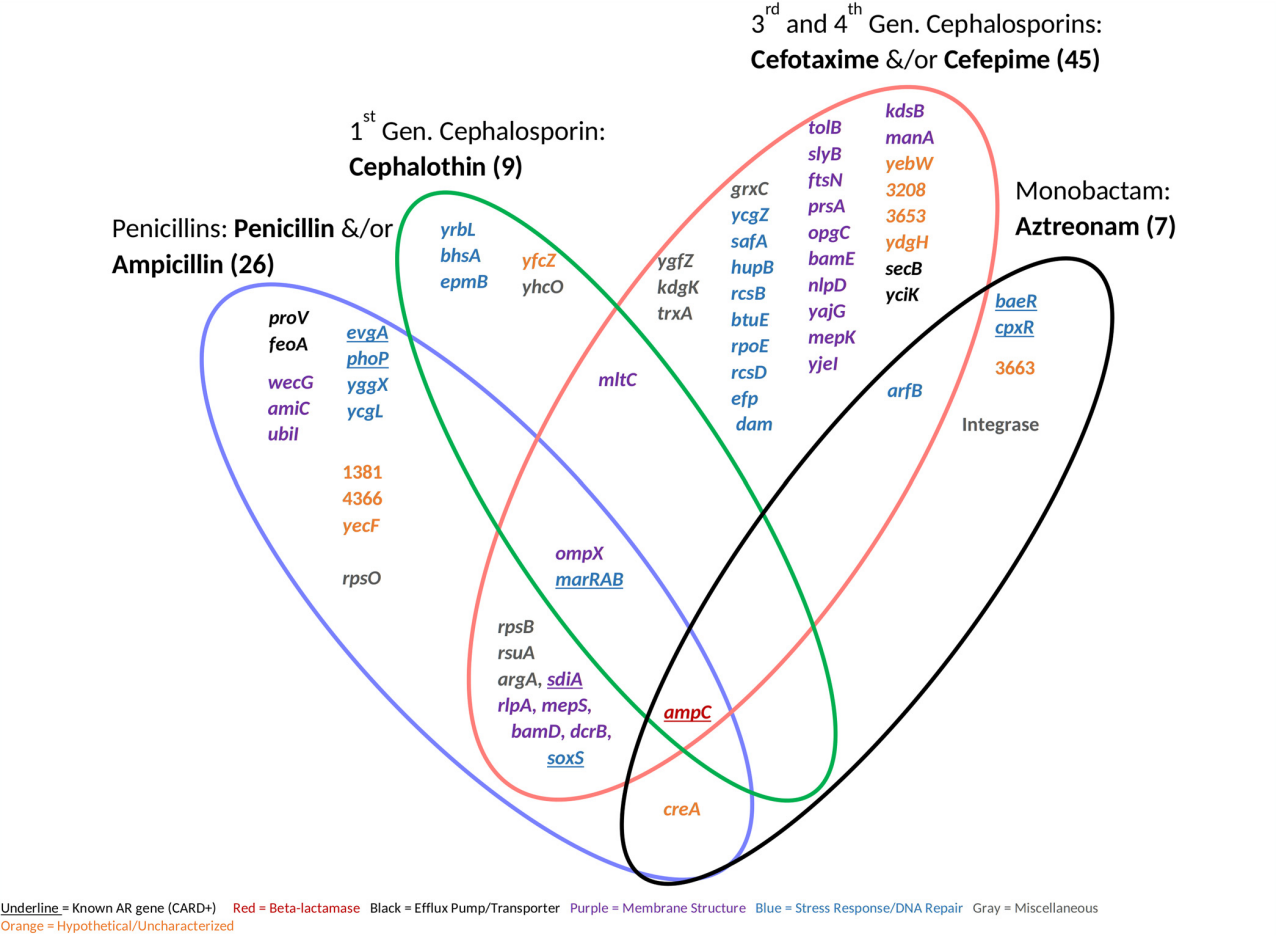
Antibiotic resistance genes shared between 6 resistance-positive beta-lactam antibiotics separated by subclass and/or generation. We identified 68 antibiotic resistance genes (shown) conferring beta-lactam resistance at the MICs. Known antibiotic resistance genes were classified using the Comprehensive Antibiotic Resistance Database by gene name.

**FIG 6:**
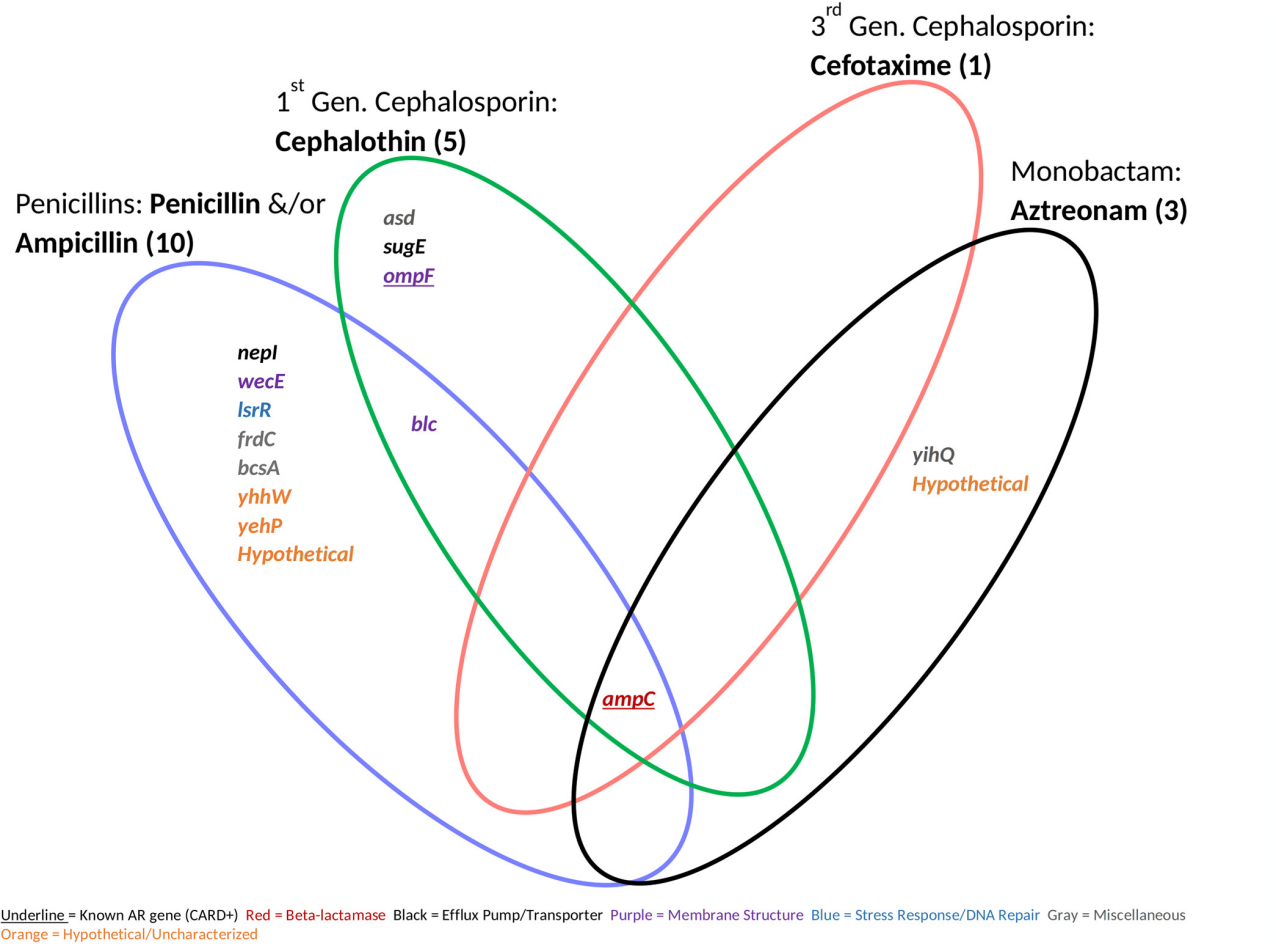
Antibiotic resistance genes conferring resistance above the MICs. Genes are shared between five resistance-positive beta-lactam antibiotics separated by subclass and/or generation. We identified 15 antibiotic resistance genes (shown). Known antibiotic resistance genes were classified using the Comprehensive Antibiotic Resistance Database by gene name.

**FIG 7:**
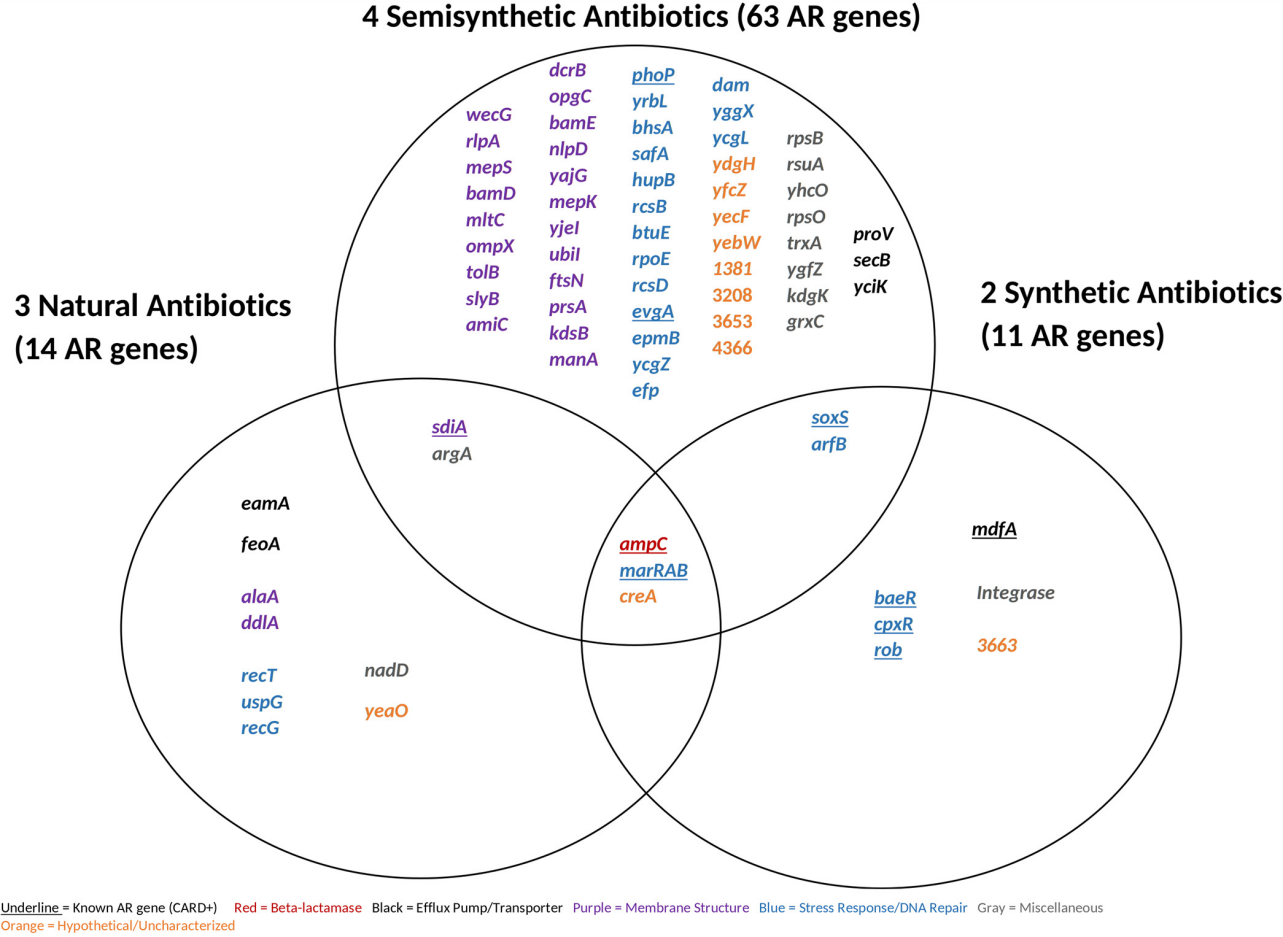
Antibiotic resistance genes shared between resistance-positive antibiotics classified by origin. We identified 78 antibiotic resistance genes causing resistance at the MICs. Known antibiotic resistance genes were classified using the Comprehensive Antibiotic Resistance Database by gene name.

**FIG 8:**
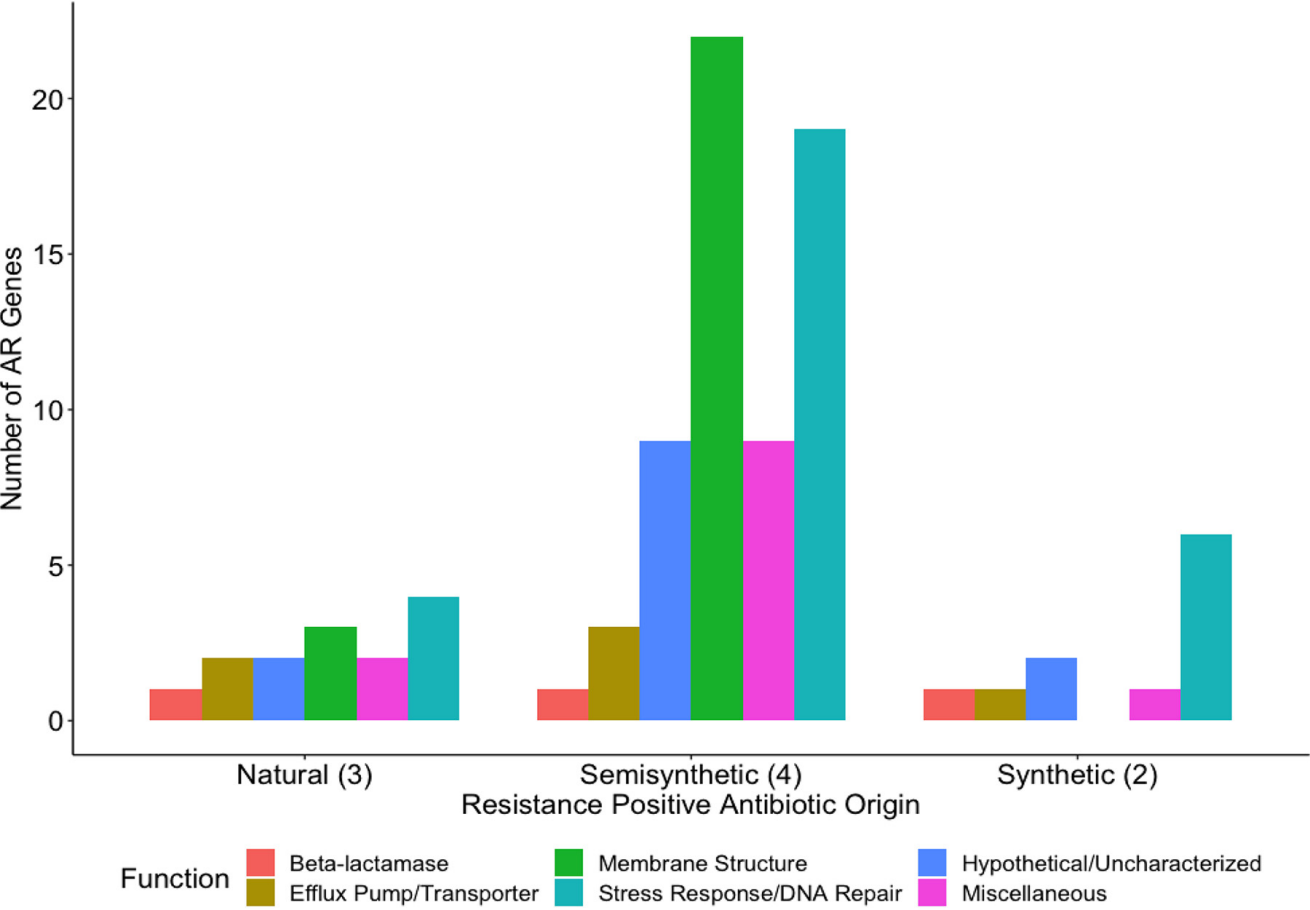
Number of antibiotic resistance genes conferring latent resistance to antibiotics at the MICs, classified by origin. The Kruskal Wallis rank sum test determined a significant difference between antibiotic origin groups (*P* < 0.05). The Dunn test was used *post hoc* to determine which pairs of groups are different. There is a significant difference in the number of antibiotic resistance genes between the semisynthetic and synthetic antibiotic groups (*P* < 0.05).

Through a gene amplification approach, we manipulated E. coli by randomly cloning genes into a high-copy vector and then reintroducing the vector into the E. coli host (Fig. 1). Utilizing a high-copy-number plasmid increases the gene copy number, thereby increasing the template for expression. AR clones were then selected by plating on 18 antibiotics spanning 8 antibiotic classes (Table 1). First, we evaluated the minimum concentration of antibiotic needed to inhibit the growth of the wild-type strain (E. cloni, a laboratory E. coli strain). These concentrations were then used to screen clones for cryptic resistance. In this study, “resistance” is in reference to the wild-type strain and means that clones were able to grow at a concentration at which the wild-type was inhibited. We subsequently tested clones for their MIC by plating transformants on antibiotic concentrations higher than the wild type’s MIC (Table 2). Plasmid inserts were sequenced from resistant clones and compared to the Comprehensive AR Database (14). We conducted a quantitative analysis of latent AR genes according to their functional categories (Fig. 2 and 3) and then qualitatively analyzed latent AR genes shared between antibiotic classes (Fig. 4 to 6). We analyzed unclassified (cryptic) AR genes and antibiotic characteristics that led to latent and cryptic antibiotic resistance. We then examined the relation between antibiotic origin (natural, semisynthetic, synthetic) and resistance (Fig. 7 and 8). Thus, we were able to systematically characterize genes that conferred an AR phenotype when amplified in E. coli.

**TABLE 1:** Total antibiotics tested and their respective properties

Biochemical property	Site of action	Class and subclass	Origin	Antibiotic (Ab)	Ab concentration^*a*^	Resistance^*b*^	No. of clones^*c*^
Hydrophilic	Cell wall	Beta-lactams					
		Penicillins	Natural	Penicillin	64	+	16
		Cephalosporins	Semisynthetic	Ampicillin	8	+	50
				Cephalothin	32	+	15
				Cefoxitin	64	−	0
				Cefotaxime	0.25	+	67
				Cefepime	0.125	+	45
		Monobactams	Synthetic	Aztreonam	0.25	+	19
		d-cycloserine	Natural	d-cycloserine	32	+	24
Amphipathic	Cytoplasmic membrane	Polymyxins	Natural	Polymyxin B	0.5	+	>1,000
Hydrophobic	Protein synthesis	Chloramphenicol	Synthetic	Chloramphenicol	8	+	16
		Aminoglycosides	Natural	Gentamicin	4	−	0
			Semisynthetic	Amikacin	16	−	0
		Tetracyclines	Natural	Tetracycline	4	−	0
			Natural	Chlortetracycline	4	−	0
			Semisynthetic	Doxycycline	4	−	0
	DNA synthesis	Fluoroquinolones	Synthetic	Nalidixic Acid	4	−	0
			Synthetic	Norfloxacin	0.125	−	0
		Nitrofurans	Synthetic	Nitrofurantoin	1	−	0

a The minimum concentration of antibiotic (μg/ml) needed to inhibit the growth of *E. cloni* cells (Lucigen). This concentration (MIC) was used to screen clones for cryptic antibiotic resistance.

b If resistance occurred in our study, this is denoted as “+”.

c The number of clones indicates the number of colonies that appeared if resistance occurred at the MIC.

**TABLE 2:** Total number of clones when testing concentrations above the MIC

Antibiotic (Ab)	Ab concentration MIC^*a*^	No. clones MIC^*b*^	No. clones 2× MIC^*b*^	No. clones 4× MIC^*b*^	No. clones 8× MIC^*b*^
Penicillin	64	16	8	0	0
Ampicillin	8	50	9	5	0
Cephalothin	32	15	4	2	0
Cefoxitin	64	0			
Cefotaxime	0.25	67	1	0	0
Cefepime	0.125	45	0	0	0
Aztreonam	0.25	19	3	0	0
d-cycloserine	32	24	0	0	0
Polymyxin B	0.5	>1,000	0	0	0
Chloramphenicol	8	16	0	0	0
Gentamicin	4	0			
Amikacin	16	0			
Tetracycline	4	0			
Chlortetracycline	4	0			
Doxycycline	4	0			
Nalidixic acid	4	0			
Norfloxacin	0.125	0			
Nitrofurantoin	1	0			

a The minimum concentration of antibiotic (μg/ml) needed to inhibit the growth of *E. cloni* cells (Lucigen). This concentration (MIC) was used to screen clones for cryptic antibiotic resistance.

b The number of clones indicates the number of colonies that appeared if transformants showed resistance at the MIC, 2× the MIC, 4× the MIC, and 8× the MIC.

Resistance occurred in response to 50% of the antibiotics tested. Antibiotics included chloramphenicol, d-cycloserine, polymyxin B, and 6 beta-lactams (Table 1). Known AR genes (i.e., CARD positive) comprised 13% of the total identified genes, whereas the majority (87%) of the identified genes were unclassified AR genes (i.e., CARD negative). Genes related to stress response and/or DNA repair conferred resistance to all resistance-positive antibiotics (Fig. 2) and were highly represented (17 to 75% of genes for each antibiotic). However, many uncharacterized or hypothetical proteins conferred resistance to each positive antibiotic except for chloramphenicol and polymyxin B. Genes from all functional categories (beta-lactamase, efflux pump/transporter, membrane structure, stress response/DNA repair, hypothetical/uncharacterized, and miscellaneous) conferred resistance to beta-lactam antibiotics (Fig. 2 and 3). On the contrary, genes from only 2 to 4 functional categories conferred resistance to chloramphenicol, d-cycloserine, and polymyxin B. Genes affecting membrane structure comprised 32% and 40% of genes conferring resistance to beta-lactams and d-cycloserine, respectively (Fig. 3). This is possibly to alleviate the stress on cell wall biosynthesis of beta-lactams and d-cycloserine (15). We observed that a wide diversity of genes conferred resistance to beta-lactams, and stress response/DNA repair genes conferred resistance to all resistance-positive antibiotics.

We next identified AR genes shared between antibiotic classes (Fig. 4). The genes conferring resistance to more than one antibiotic class (multiple beta-lactams and chloramphenicol) when amplified were *soxS* and those in the *marRAB* operon. These are known AR genes that encode transcriptional regulators for general stress responses (16). When overexpressed, they may activate the multidrug efflux pump AcrAB and decrease expression of porin OmpF to decrease cell permeability. In contrast, there were no unclassified AR genes that conferred resistance to more than one antibiotic class, suggesting that cryptic antibiotic resistance may stem from a certain gene response specific to the antimicrobial mechanism of action.

We uncovered a diversity of previously unclassified AR genes (CARD negative) that conferred cryptic resistance to d-cycloserine or polymyxin B (Fig. 4). The *alaA* and *ddlA* genes, which encode glutamate-pyruvate aminotransferase and d-alanine-d-alanine ligase A, respectively, conferred resistance to d-cycloserine when amplified in our study. Inhibiting the biosynthesis of amino acids integrated within the Gram-negative peptidoglycan peptide stem has been investigated as a putative approach for novel antibiotics (17), and *alaA* and *ddlA* hold an important role for L-alanine and d-alanine synthesis, respectively, in E. coli. *ddlA* has long been known to be the target gene for d-cycloserine (18). This was the only case in which gene amplification of the antibiotic target gene conferred latent resistance in our study. Similarly, this occurred to only 4 out of 31 antibiotics in a previous study observing for latent antibiotic resistance (5). Overexpression of target genes occurs primarily to antimicrobial agents that act on a single target gene. This is less common for Gram-negative antibiotics, as they usually inhibit a family of related enzymes or act on nonprotein targets, such as the cytoplasmic membrane (19). For example, many beta-lactams bind multiple targets (penicillin-binding proteins), which catalyze peptidoglycan cross-linking; polymyxins disrupt the integrity of the cytoplasmic membrane. Here, *eamA* (previously named *ydeD*) also conferred d-cycloserine resistance. Although this is an unclassified AR gene we have identified in this study, EamA is an exporter classified within the drug/metabolite transporter superfamily (20). A high copy number of DNA repair proteins RecT and RecG conferred resistance to d-cycloserine and polymyxin B, respectively, in our study. RecT has not been previously linked to resistance to our knowledge, but RecG has been shown to decrease polymyxin B susceptibility when upregulated in P. aeruginosa (21). Here, universal stress protein G, UspG, also conferred resistance to polymyxin B, and it has previously been shown to be regulated during colistin (a polymyxin drug) treatment in E. coli (22). *nadD*, which encodes an essential enzyme involved in both the *de novo* biosynthesis and salvage of NAD^+^ and NADPH (23), also conferred resistance to polymyxin B in our assay. This gene has been shown to be a promising antimicrobial target with broad-spectrum activity (23). No unknown AR genes conferred resistance to chloramphenicol in our assay. Genes within the stress response/DNA repair functional category have a broad AR potential, as they conferred cryptic resistance to nearly all positive antibiotics (Fig. 4 and 5). Even though some of the genes identified have previously been linked to antibiotic resistance, they have not been established as the culprit of resistance. This assay demonstrates that these unknown genes conferred an AR phenotype when present in high copy number.

We also found a diversity of unknown AR genes that conferred cryptic resistance to beta-lactam antibiotics (Fig. 4 and 5). The majority (6 out of 11) of the unclassified AR genes that conferred resistance to multiple generations of beta-lactams had functions related to membrane structure. Three genes (*rlpA* [24], *mepS* [25], and *mltC* [24]) were related to cell wall/peptidoglycan recycling. Even though these genes are not antibiotic targets, *rlpA* is directly upstream of *dacA*, which encodes penicillin-binding protein 5 (Pbp5) (26). A Pbp5 associated protein has shown to increase cephalosporin resistance when overexpressed in Enterococcus faecium (27). Therefore, genes associated with or in close proximity to Pbp5 may be capable of conferring cryptic resistance when amplified. Outer membrane protein X, encoded by *ompX*, also conferred resistance to multiple beta-lactams in our assay. Overexpression of *ompX* can repress expression of OmpC and OmpF porins and lead to a decreased susceptibility to beta-lactams (28). We also saw that genes related to maintaining cell membrane permeability and integrity conferred resistance to the penicillins and cephalosporins (Fig. 5). These proteins included WecG (29), UbiI (30), TolB (31), SlyB (32), PrsA (33), OpgC (34), KdsB (35), ManA (36), AmiC (37), NlpD (37), YajG (38), and MepK (39). The latter four proteins are involved in cell wall synthesis and recycling. We found that at least one stress response gene conferred resistance to each beta-lactam antibiotic, and many of these genes were associated with a global stress response and/or two-component regulatory systems. For example, *ycgL*, which conferred resistance to the penicillins in our assay, is a gene that is potentially regulated by SOS, a global response to DNA damage (40, 41). Similarly, *yrbL*, which conferred resistance to cephalothin, is regulated by PhoP, a part of a two-component system that senses and responds to a variety of environmental changes (42, 43). The PhoP/PhoQ system is activated by *safA* (44), which conferred resistance to cefotaxime in our study. The PhoP/PhoQ system is connected to the EvgS/EvgA two-component system, and *safA*, the “connector,” connects these two systems (45). Here, a high copy number of YcgZ led to cefepime resistance, and this protein interacts with the Rcs two-component regulatory system while being regulated by *marA* (46). The Rcs system consists of the response regulator RcsB and phosphotransferase RcsD (47), and these proteins conferred resistance to cefotaxime and cefepime, respectively, in our assay. *creA*, which conferred resistance to multiple beta-lactams here, has an uncharacterized function, but it is adjacent to the CreBC two-component regulatory system (48). We saw that more hypothetical proteins conferred beta-lactam resistance than unclassified AR efflux pumps/transporters when amplified. The low number of efflux pumps/transporters causing latent resistance could be due to the antibiotic target site and cell structure (49, 50). Beta-lactams do not need to cross the cytoplasmic membrane to reach their target and thereby face the Gram-negative cell wall as their primary barrier. We found that beta-lactam antibiotic resistance was dominated by genes related to the cell wall and general stress transcriptional regulators.

We observed more genes conferring latent resistance to semisynthetic antibiotics than to natural or synthetic antibiotics (Fig. 7 and 8). There were more semisynthetic-resistant-positive antibiotics (4) than natural-resistant-positive (3) or synthetic-resistant-positive (2) antibiotics. There is a significant difference between the antibiotic origin groups (*P* < 0.05) driven by a difference between the semisynthetic and synthetic antibiotic groups (*P* < 0.05). We had predicted that latent resistance would be less common in the presence of synthetic antibiotics due to their stronger activity, but this was not the case. Semisynthetic antimicrobials are generally made to act against bacteria that developed resistance to the prior generation (51), suggesting that semisynthetic antibiotics can be specifically optimized to prevent resistance. E. coli may be more capable of developing latent resistance to semisynthetic antibiotics, specifically beta-lactams, as the resistance-positive semisynthetic antibiotics consisted of cephalosporins. A total of 35% of genes conferring resistance to semisynthetic antibiotics were related to membrane structure. This is likely to lessen the impact of cephalosporins on cell wall biosynthesis. Cell membrane-related genes did not confer latent resistance to synthetic antibiotics in our study even though a beta-lactam was present (Fig. 7 and 8). This potentially indicates that synthetic antibiotics can overcome the effect of highly amplified cell membrane genes. A total of 29 to 55% of AR genes for all three origins were related to stress response/DNA repair, highlighting the broad AR potential for this functional category. This study did not determine a link between antibiotic origin and latent resistance, as most antibiotics from each origin were beta-lactams, showing a stronger link between antibiotic mechanism of action and latent resistance.

Certain antibiotic characteristics may contribute to cryptic and/or latent antibiotic resistance. Resistance occurred to nearly all beta-lactams tested, chloramphenicol, d-cycloserine, and polymyxin B. Beta-lactams (49) and d-cycloserine (52, 53) are hydrophilic and inhibit cell wall biosynthesis. Polymyxin B inhibits the cytoplasmic membrane and is amphipathic (54). The Gram-negative bacteria outer membrane acts a first defense mechanism against antibiotics due to the hydrophobic lipid bilayer and specifically sized aqueous pores (49, 50, 55). Antibiotics can penetrate the outer membrane by dissolving in the lipid bilayer or crossing through the pores, the hydrophobic or hydrophilic mechanism, respectively (49, 50). Antibiotics with targets on the outer surface of the cytoplasmic membrane (exposed) need to cross the lipid matrix, facing the outer membrane barrier (50). Beta-lactams and polymyxin B have exposed targets, while d-cycloserine, although hydrophilic, needs to permeate the outer and cytoplasmic membranes to reach its target. Hydrophobic antibiotics usually need to penetrate the outer and cytoplasmic membranes since their target is generally involved with DNA or protein synthesis (50). Therefore, it may be biochemically simpler for E. coli to inhibit antibiotics with hydrophilic properties as opposed to hydrophobic antibiotics. Resistance did not occur in the presence of nitrofurans, fluoroquinolones, tetracyclines, and aminoglycosides, which are all hydrophobic (Table 1) and need to cross the cytoplasmic membrane to reach their target (50, 56, 57). Resistance also occurred to chloramphenicol, which is noteworthy because it is now synthetically made and hydrophobic (58), but only known AR genes conferred resistance to it in our study (Fig. 4). We found that beta-lactams, hydrophilic antibiotics, and antibiotics that inhibit the cell wall or cytoplasmic membrane were more likely to induce latent resistance in E. coli.

We captured many known AR genes (Fig. 4), suggesting that this was a robust approach to examine the effect of gene amplification on latent resistance profiles. Gene amplification of *ampC* conferred latent and higher ranges of resistance to all beta-lactams except for cefoxitin and cefepime (Fig. 5 and 6), second- and fourth-generation cephalosporins, respectively (59). There may have been no resistance to cefoxitin, as it is stable against *ampC* activity and cefepime is a weak substrate for *ampC* (60). This gene is encoded on the chromosomes of many *Enterobacteriaceae*, but it is commonly weakly expressed (60). *sdiA*, which encodes a cell division regulator and activates AcrAB multidrug efflux pump (61), conferred resistance to multiple generations of beta-lactams in our assay (Fig. 5). Gene amplification of the two-component regulatory systems BaeSR, CpxAR, EvgAS, and PhoPQ also conferred resistance to multiple beta-lactams. Two-component systems, which activate responses to environmental stress, are known to increase antibiotic resistance via several mechanisms, including upregulation of multidrug efflux pumps and changes in cell permeability (62). A multidrug efflux pump, MdfA, conferred resistance to chloramphenicol in our study (Fig. 4). MdfA was originally classified as the CmIA/Cmr chloramphenicol exporter (63), further validating the chloramphenicol resistance phenotype. We observed that the amplification of *soxS*, *rob*, and genes from the MarRAB operon conferred resistance to several beta-lactams and/or chloramphenicol. These genes encode transcriptional regulators for general stress signals such as oxidative stress, acidic pH, and antibiotics (16). When upregulated, they may activate the multidrug efflux pump AcrAB and repress expression of porin OmpF to decrease cell permeability (16). However, high copy numbers of *soxS*, *rob*, and genes from the MarRAB operon did not confer resistance above the MIC in this study (Fig. 6), showing that these genes are limited in their resistance potential. The identification of known AR genes validated this method as an effective way to test for latent resistance genes in a high-throughput manner.

A caveat of this study is that our assay cannot discern multiple or complex gene regulation. First, this assay is unable to capture mechanisms, whereby two genes are required for resistance but are not colocated. Second, this assay did not distinguish coregulation that occurred between genes present on the plasmid insert. In these cases, we took a probabilistic approach and called the gene with the highest coverage the putative AR gene. Even though coverage varied across antibiotics for some genes, we chose the gene with the highest coverage to maintain consistency and accuracy. Opting for smaller insert sizes (1 to 2 kb) may cause less cooccurrence of genes. Due to the high frequency (81%), most gene calls were subject to cooccurrence, but choosing the gene with the highest coverage ensures that the most probable AR gene was identified. Last, it is also a caveat that this study cannot yet be interpreted in a clinical sense, as our MIC methodology is not clinically standard. We needed to replicate the approach (LB agar plate) that we used to screen clones for cryptic antibiotic resistance, as our main goal was to demonstrate the biological mechanism more than the clinical relevance. Our study demonstrates that E. coli is capable of increasing the concentration of antibiotic in which it can grow. Translation for a clinical setting would require further examination of the inhibition concentrations using a clinical standard.

A diverse repertoire of latent AR genes may be a widespread phenomenon among bacteria. Microbiomes from humans (64), sea gulls (65), soil (66, 67), river (68), and ocean water (69) have shown to be reservoirs of diverse known and unknown AR genes. Even though these functional metagenomic assays were used to survey AR genes in a certain environment, this technique can also be used to identify silent resistance genes which are capable of conferring resistance when amplified in other hosts but not in their native context (70). Thus, the presence of cryptic genes activated by gene amplification may be a widespread phenomenon. However, the use of a surrogate host to identify resistance genes can confound results, as phenotypic resistance in donor strains may not translate to resistance in the native genomic context. Therefore, we have developed an assay that circumvents this limitation and expresses genes in the organism of interest. The diversity of microbes, which appears to be the principle of latent resistance, suggests that this could be important for the emergence of resistance to antibiotics. As this platform is used on other pathogens, a predictive model could be built to classify types of antibiotics and organisms that are less likely to promote latent resistance while also identifying novel antibiotic resistance genes.

## MATERIALS AND METHODS

### Strain, media, and culture conditions.

*E. cloni* 10G Supreme cells (Lucigen, Middleton, WI, USA), the wild-type strain, were grown in Luria-Bertani (LB) medium and incubated overnight at 37°C unless otherwise stated.

### Resistance profile.

To appropriately screen clones for cryptic antibiotic resistance, the minimum concentration of antibiotic needed to inhibit the growth of 10^6^ *E. cloni* cells was determined for all antibiotics (Table 1) using LB agar plates. The listed antibiotics were tested to include a range of classes (mechanisms of action) and origins (natural, semisynthetic, or synthetic) if available. The range of concentrations tested for each antibiotic was 0.032 to 512 μg/ml. Growth was identified as more than 10 colonies. The lowest concentration that led to no growth on 2 out of 3 replicates was used to screen clones for cryptic antibiotic resistance.

### Cloning and screening.

Genomic DNA was extracted from *E. cloni* cells using the Wizard Genomic DNA purification kit (Promega Corporation, Madison, WI, USA). At least 10 μg of genomic DNA were sheared to a target size of 2 kb using a Covaris S220 focus acoustic shearer (Covaris Inc., Woburn, MA, USA). Fragments of 1 to 3 kb were extracted from a 1% agarose gel using the Zymoclean gel DNA recovery kit (Zymo Research, Irvine, CA, USA). DNA was treated with the NEBNext end repair module to create blunt ends on the fragmented DNA (New England Biolabs, Ipswich, MA, USA). The end-repaired DNA was purified using the DNA Clean and Concentrator-10 kit (Zymo Research). DNA was ligated into pSMART-HCKan vector (accession number AF532107) and then electroporated into *E. cloni* cells as per the CloneSmart Blunt cloning kit (Lucigen). This vector relies on endogenous promoters. A vector background control and a positive-control insert DNA (HincII-digested lambda DNA) were processed as well to determine the ligation and transformation efficiencies. Transformed cells were recovered at 37°C for 1 h. Cultures were then diluted 1:10 and 1:100, and 100 μl of each was plated on LB Lennox agar containing kanamycin (30 μg/ml) to determine the total CFU and the number of plasmids tested on each antibiotic. Totals of 50 μl of the vector background control and 5 μl of the positive-control insert DNA were plated on LB Lennox kanamycin agar plates.

To test for cryptic antibiotic resistance, 150 μl of undiluted recovered transformants was plated on LB Lennox kanamycin agar containing one of one of 18 antibiotics (Table 1). After overnight incubation, resistant transformants were pooled for each antibiotic using 1 to 2 ml of phosphate-buffered saline (PBS). Prior to pooling, 2 colonies from each plate were restreaked onto agar containing the same antibiotics to confirm resistant clones. Pooled plasmid DNA was extracted from each PBS suspension (9 total samples, 1 from each resistance-positive antibiotic) using the ZR plasmid miniprep kit (Zymo Research) and stored at –20°C. Plasmid inserts containing the AR genes were amplified via PCR. This PCR used 25 μl reactions, including 12.5 μl of AccuStart II PCR SuperMix 2× (Quantabio), 3 μl (1.5 ng) of plasmid DNA, 4.5 μl of nuclease-free water, and 2.5 μl of SL1 and SR2 primers (Lucigen). The reaction cycle conditions follow those delineated for AccuStart II PCR SuperMix 2× (Quantabio). PCR products were purified using the QIAquick PCR purification kit (Qiagen).

The cloning was repeated to obtain the AR clones’ MICs and the range of resistance conferred by gene amplification. A total of 150 μl of undiluted recovered transformants was plated on concentrations 2, 4, and 8 times the MIC (Table 2). This was done for the 9 resistance-positive antibiotics (Table 1). All clones grown above the MIC were restreaked onto agar containing the same antibiotics. Then, these restreaked clones were grown in LB broth containing the same antibiotics and incubated overnight. Plasmid DNA was individually extracted from each culture (1 from each AR clone) using the ZR plasmid miniprep kit (Zymo Research) and stored at –20°C.

### Library preparation, sequencing, and analysis.

For pooled plasmids, library preparation was performed according to the PCR barcoding genomic DNA (SQK-LSK109) protocol for the MinION device (Oxford Nanopore Technologies). A total of 200 fmol of each library was end-prepped for ligation with barcode adaptors using the NEBNext Ultra II end-repair/dA-tailing module (New England Biolabs). DNA samples were purified using 1× volume AMPure XP beads (Beckman Coulter). Barcode adapters (Oxford Nanopore Technologies) were ligated onto the end-prepped DNA libraries using the Blunt/TA ligase master mix (New England Biolabs). After bead-cleaning DNA libraries, barcodes from PCR barcoding expansion 1 to 12 (Oxford Nanopore Technologies) were added onto the samples via PCR using LongAmp *Taq* 2× master mix (New England Biolabs). Barcoded libraries were bead purified and equimolar pooled. The pooled libraries were end-prepped for ligation of sequencing adaptors and subsequently purified using beads. Sequencing adaptors were ligated onto the end-prepped DNA using ligation buffer (Oxford Nanopore Technologies), NEBNext Quick T4 DNA ligase (New England Biolabs), and adapter mix (Oxford Nanopore Technologies). The reaction mix was bead purified and quantified using the Invitrogen Qubit fluorimeter (Thermo Fisher Scientific).

Sequencing was done on the MinION flow cell (FLO-Min106 R9.4.1 version; Oxford Nanopore Technologies) using the MinION device (Mk1B version). A platform QC and priming were done on the flow cell prior to sequencing according to the manufacturer’s instructions. The final library, mixed with sequencing buffer and loading beads (Oxford Nanopore Technologies), was added to the flow cell via the SpotON sample port.

Base calling was done in real time using MinKNOW software (Oxford Nanopore Technologies) on a local computer. The sequencing run was carried out for 15 h, and the barcoded base-called reads were subsequently demultiplexed and analyzed using the “Barcoding” workflow on the EPI2ME Desktop Agent software. Demultiplexed reads were aligned and mapped to the E. coli reference genome (Lucigen) using Bowtie 2 (71). Mapped reads were assembled and processed with Anvi’o (72), which provided coverage, identity, and location within the reference strain for each aligned gene.

We chose genes that had coverage within the 99% confidence interval as putative resistance genes, which totaled 174 individual genes (292 total, taking into account repetition) across all resistance-positive antibiotics. Gene identities were confirmed with NCBI BLASTx, and gene names present within the Comprehensive AR Database (14) were identified as known AR genes. For each resistance-positive antibiotic, we identified the gene with the highest coverage as the most probable resistance gene when multiple genes were located within close proximity respective to the reference strain. After taking this into account, we found a total of 78 AR genes for the analysis (Fig. 3). A total of 81% of the 78 AR genes were associated with at least one other gene, demonstrating that the majority of plasmid inserts harbored more than one gene.

For individual plasmid extractions, Sanger sequencing was used to identify genes causing resistance at concentrations above the MIC. SL1 and SR2 primers (Lucigen) were used at 5 μM for sequencing. Gene identities were confirmed with NCBI BLASTx, and gene names present within the Comprehensive AR Database (14) were identified as known AR genes. Sequencing yielded 2 genes for each plasmid extraction, totaling 18 unique genes. If a beta-lactamase gene was either of the 2 genes, we identified the beta-lactamase as the most probable resistance gene. After taking this into account, we found 15 AR genes for the analysis (Fig. 6).
